# Numb contributes to renal fibrosis by promoting tubular epithelial cell cycle arrest at G2/M

**DOI:** 10.18632/oncotarget.8238

**Published:** 2016-03-21

**Authors:** Fengxin Zhu, Wei Liu, Tang Li, Jiao Wan, Jianwei Tian, Zhanmei Zhou, Hao Li, Youhua Liu, Fan Fan Hou, Jing Nie

**Affiliations:** ^1^ State Key Laboratory of Organ Failure Research, National Clinical Research Center of Kidney Disease, Division of Nephrology, Nanfang Hospital, Southern Medical University, Guangzhou, P.R. China; ^2^ Department of Nephrology, Guangdong General Hospital, Guangdong Academy of Medical Sciences, Guangdong Geriatric Institute, Guangzhou, P.R. China; ^3^ The VIP Medical Center, The Third Affiliated Hospital of Sun Yat-Sen University, Guangzhou, P.R. China

**Keywords:** Numb, G2/M arrest, p53, proximal tubular cells, interstitial fibrosis

## Abstract

Numb is a multifunctional protein involved in diverse cellular processes. However, the function of Numb in kidney remains unclear. Here, we reported that Numb is expressed in renal tubules and glomeruli in normal adult kidney. Numb expression was upregulated in fibrotic kidneys induced by unilateral ureteral obstruction (UUO) in mice as well as in human fibrotic kidney tissues. Numb overexpression in cultured proximal tubular cells increased the G2/M cell population and upregulated the expression of TGF-β1 and CTGF. Whereas, proximal tubule Numb knockout (PEPCK-Numb-KO) mice showed reduced G2/M arrest, decreased expression of TGF-β1 and CTGF, and attenuated fibrotic lesions due to either UUO or unilateral ischemia reperfusion nephropathy. Inhibiting p53 activity by pifithrin-β dramatically mitigated Numb-induced G2/M arrest, indicating that Numb potentiates G2/M arrest via stabilizing p53 protein. Together, these data suggest that Numb is a potential target for anti-fibrosis therapy.

## INTRODUCTION

Progressive tubulointerstitial fibrosis (TIF) is the final common pathway leading to end stage renal disease, irrespective of the initial cause [1–3]. Unfortunately, approved treatment specifically targeted to TIF is almost nonexistent. In this context, understanding the cellular mechanisms that facilitates TIF is crucial for facilitating the development of effective treatments.

Tubular epithelium is the major constituent of renal parenchyma and its contribution to the progression of fibrogenesis has been extensively studied. It has been shown that the activation of NF-κB inflammatory signaling in tubular epithelial cells (TECs) triggers the production of inflammatory chemokines and cytokines, promotes interstitial inflammation [4]. Sustained activation of ILK [5], Wnt/β-catenin [6, 7], Notch [8, 9] or hypoxia-inducible factor 1 signaling [10, 11] in TECs facilitates renal fibrosis. Most recent studies demonstrate that partial epithelial to mesenchymal transition (EMT) of TECs is activated in the event of renal damage, and these damaged TECs relays signals to promote myofibroblast differentiation and fibrogenesis [12]. Yang *et al*. reported that prolonged G2/M arrest of proximal tubular cells leads to overproduction of transform growth factor-β1 (TGF-β1) and connective tissue growth factor (CTGF) [13]. Moreover, genome-wide unbiased transcript analysis of human kidney samples revealed that defective fatty acid oxidation in TECs contributes to the development of TIF [14]. Altogether, these findings suggest that TECs play a crucial role in the pathogenesis of TIF.

Numb was originally identified as an intrinsic cell fate determinant during peripheral and central nervous system development in *Drosophila* by directing asymmetric division of progenitor cells [15, 16]. Numb and Numblike (Numbl) are mammalian homologues of *Drosophila* Numb sharing extensive sequence identity with functional redundancy [17]. In mammalian cells, Numb mediates asymmetric division, endocytosis and recycling of transmembrane proteins, and cell migration [18]. In addition, Numb has been shown to behave as a tumor suppressor through stabilization of p53 and promoting Notch and Gli1 degradation [19, 20]. However, the role of Numb in kidney and renal injury remains largely unknown.

In the current study, we found that the expression of Numb was dramatically increased in fibrotic kidney induced by unilateral ureteral obstruction (UUO) and human fibrotic kidney. To explore the role of Numb in TIF, we generated a conditional knockout mouse model in which Numb is selectively ablated from proximal tubules (PEPCK-Numb-KO). PEPCK-Numb-KO mice displayed attenuated TIF, which is correlated with a marked reduction of G2/M arrest of proximal tubular cells. Our results suggest that tubular Numb is a novel mediator of TIF.

## RESULTS

### Induction of Numb in mouse model of obstructive nephropathy

**Figure 1:**
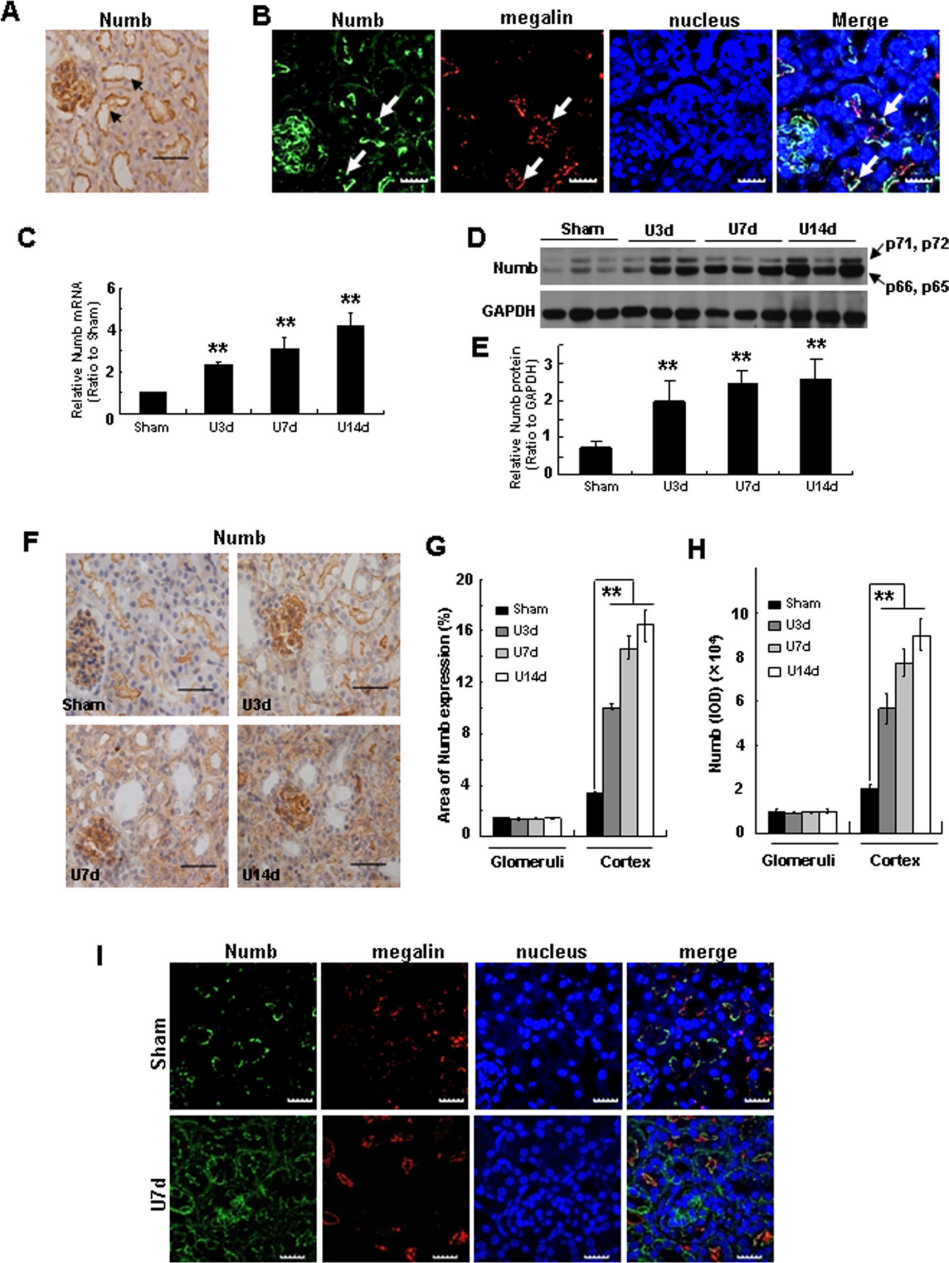
Numb expression is induced in TECs after obstructive injury **A.** Immunohistochemistry staining by using anti-Numb antibody shows the abundance and distribution of Numb protein in the kidney of C57BL/6J mice at the age of 8-12 weeks (see arrows). Bar=50μm. **B.** Immunofluorescence staining shows the co-localization of Numb (green) and megalin (red) in proximal tubules (see arrows). Nuclei were stained with DAPI (blue). Images were taken by confocal microscopy. Bar=20μm. **C.** Real time-PCR shows the level of Numb mRNA in injured kidney was increased in a time-dependent manner after UUO. C57BL/6J mice were subjected to UUO, and kidney tissues were collected at different time points after surgery as indicated. Relative Numb mRNA levels were expressed as fold induction over sham controls after normalization with GAPDH. **D.** Western blot analysis shows the induction of Numb protein in fibrotic kidney induced by UUO. **E.** Graphic representation of relative protein level of Numb normalized to GADPH. **F.** Immunohistochemistry staining shows the expression and distribution of Numb in the kidney at day 3, 7 and 14 after UUO. Bar=50μm. Quantification of the percentage of the Numb-positive area **G.** and the IOD of Numb **H.** in kidney sections. **I.** Immunofluorescence staining shows the co-staining of Numb (green) with megalin (red) in UUO. Bar=20μm. Data are expressed as mean±SD, n=6. **p<0.01 versus sham.

We first performed immunohistochemistry staining to examine the distribution and expression of Numb in normal adult kidney of C57BL/6J mice. As shown in Figure 1A, a strong Numb signal was detected at the apical side of renal tubules as well as glomeruli. To further decipher its localization in TECs, Numb was co-stained with megalin, a marker of proximal tubules, indicating that Numb is expressed in proximal tubules (Figure 1B).

To investigate the potential relevance of Numb with TIF, we examined Numb expression in mouse model of obstructive nephropathy induced by UUO. As shown in Figure 1C-1E, real time PCR and Western blotting demonstrated that both the mRNA and protein levels of Numb were significantly induced in fibrotic kidney in a time-dependent manner. The mammalian Numb gene is alternatively spliced, producing four isoforms with molecular masses of 65, 66, 71 and 72 kDa. As shown in Figure 1D, the densities of the upper band which is for p72 and p71 isoforms, and the lower band which is for p66 and p65 isoforms, were both enhanced, suggesting that the expression of all four isoforms of Numb was induced in fibrotic kidney. Immunohisotchemistry staining revealed that both the intensity and the positive area of Numb staining were significantly increased in tubules after UUO, which might because UUO mainly induces tubular cell injury (Figure 1F-1H). In addition, Numb was translocated to the basal lateral membrane in injured tubules. Co-staining Numb with megalin demonstrated that, in accordance with that in normal kidney, increased Numb was also detected in proximal tubules after UUO (Figure 1I).

### Generation of mice with renal proximal tubule-specific ablation of Numb

**Figure 2:**
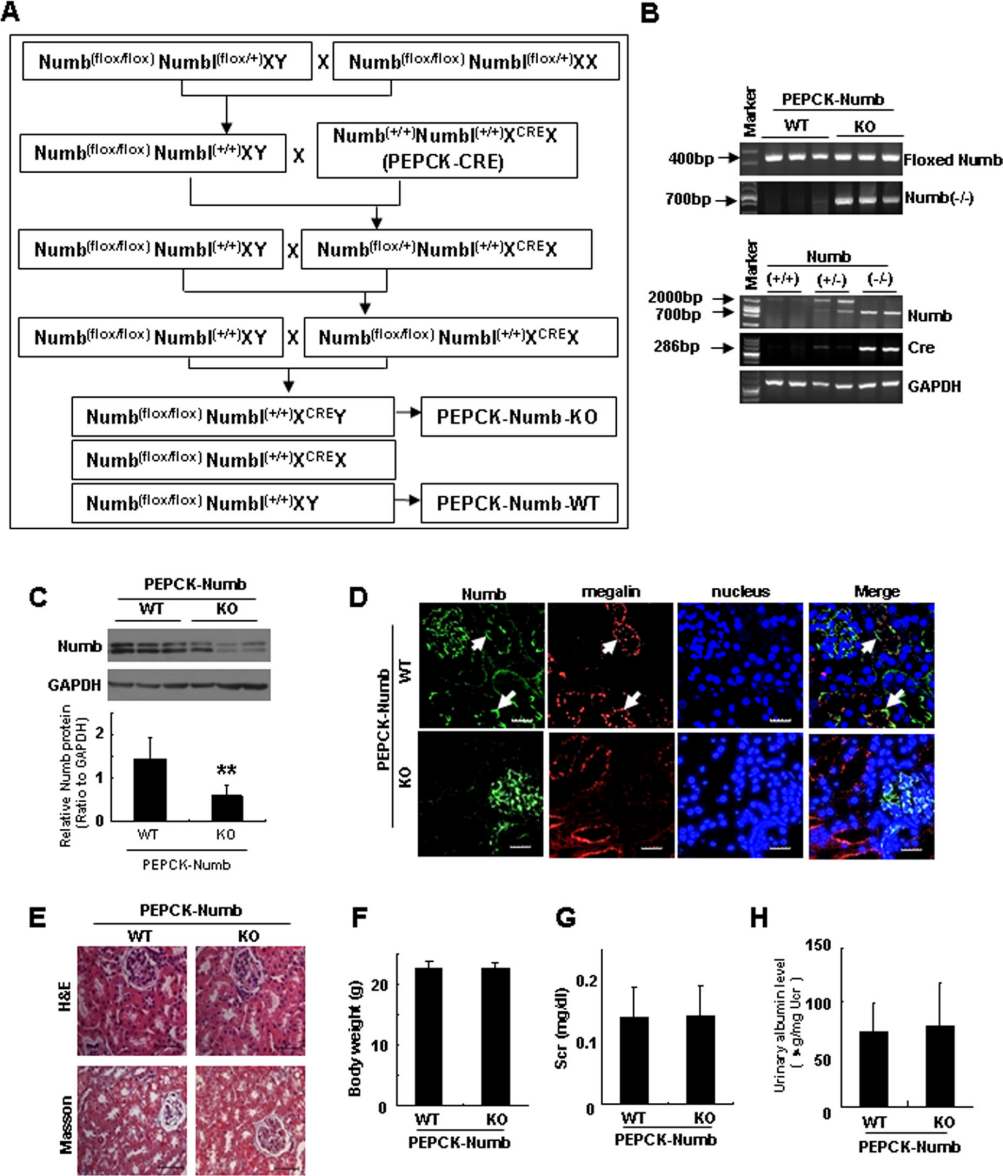
Mouse model of targeted deletion of Numb from renal proximal tubules **A.** Breeding protocol for the production of proximal tubule-specific Numb knockout mice (PEPCK-Numb-KO). Male littermate mice with confirmed genotypes were used for experiments. Age and sex-matched Numb^flox/flox^Numblike^+/+^ mice from the same litters were used as control mice (PEPCK-Numb-WT). **B.** PCR-based genotyping to confirm Numb deletion from renal cortical tissues. Genomic DNA was isolated from renal cortical tissues of PEPCK-Numb-KO and PEPCK-Numb-WT. The DNA samples (50ng per lane) were PCR-amplified using the primer pairs described in the methods section. **C.** Renal cortical tissues are dissected from kidneys of PEPCK-Numb-KO and PEPCK-Numb-WT mice for homogenization to collect whole-tissue lysates for immunoblotting of Numb. GAPDH was used to verify equivalent loading. **p<0.01 versus control mice. **D.** Immunofluorescence staining analysis of Numb expression in PEPCK-Numb-KO mice. Renal cortex sections of PEPCK-Numb-KO and PEPCK-Numb-WT mice were paraffin-fixed and then co-stained with antibody against Numb (green) or megalin (red) (see arrows). Nuclei were stained with DAPI. Bar=20μm. **E.** Representative micrographs show the morphology of PEPCK-Numb-WT and PEPCK-Numb-KO kidneys by H&E (upper panel) and Masson trichrome staining (lower panel). Bar=50μm. (F-H) There were no differences in body weight **F.** serum creatinine **G.** and urinary albumin level **H.** between PEPCK-Numb-WT and PEPCK-Numb-KO mice (n=6). Scr, serum creatinine. Ucr, urine creatinine.

It has been reported that Numb and Numbl play redundant roles in various mammalian tissues [21–23]. To determine the role of tubular-Numb in TIF, we generated a conditional knockout mouse in which Numb gene was ablated from proximal tubules. As shown in Figure 2A, Numb^flox/flox^Numblike^+/+^ mice were intercrossed to transgenic mice expressing the Cre recombinase under the control of a modified PEPCK promoter to generate Numb^flox/flox^Numblike^+/+^X^cre^Y mice with PEPCK-Cre mediated Numb deletion from proximal tubules (PEPCK-Numb-KO). The genotype of PEPCK-Numb-KO was determined by PCR (Figure 2B). Western blotting showed that Numb expression was dramatically reduced in PEPCK-Numb-KO mice compared to their wild-type littermates (Figure 2C). Co-staining Numb with megalin revealed a loss of Numb protein in proximal tubules in PEPCK-Numb-KO mice (Figure 2D). Hematoxylin and eosin (H&E) and Masson trichrome staining showed no appreciable abnormality in kidney morphology in PEPCK-Numb-KO mice at the age of 8-10 weeks (Figure 2E). Moreover, there were no detectable differences in body weight, serum creatinine and urinary albumin between PEPCK-Numb-KO and their wild-type littermates (Figure 2F-2H).

### Deletion of Numb from proximal tubules attenuates renal fibrosis

**Figure 3:**
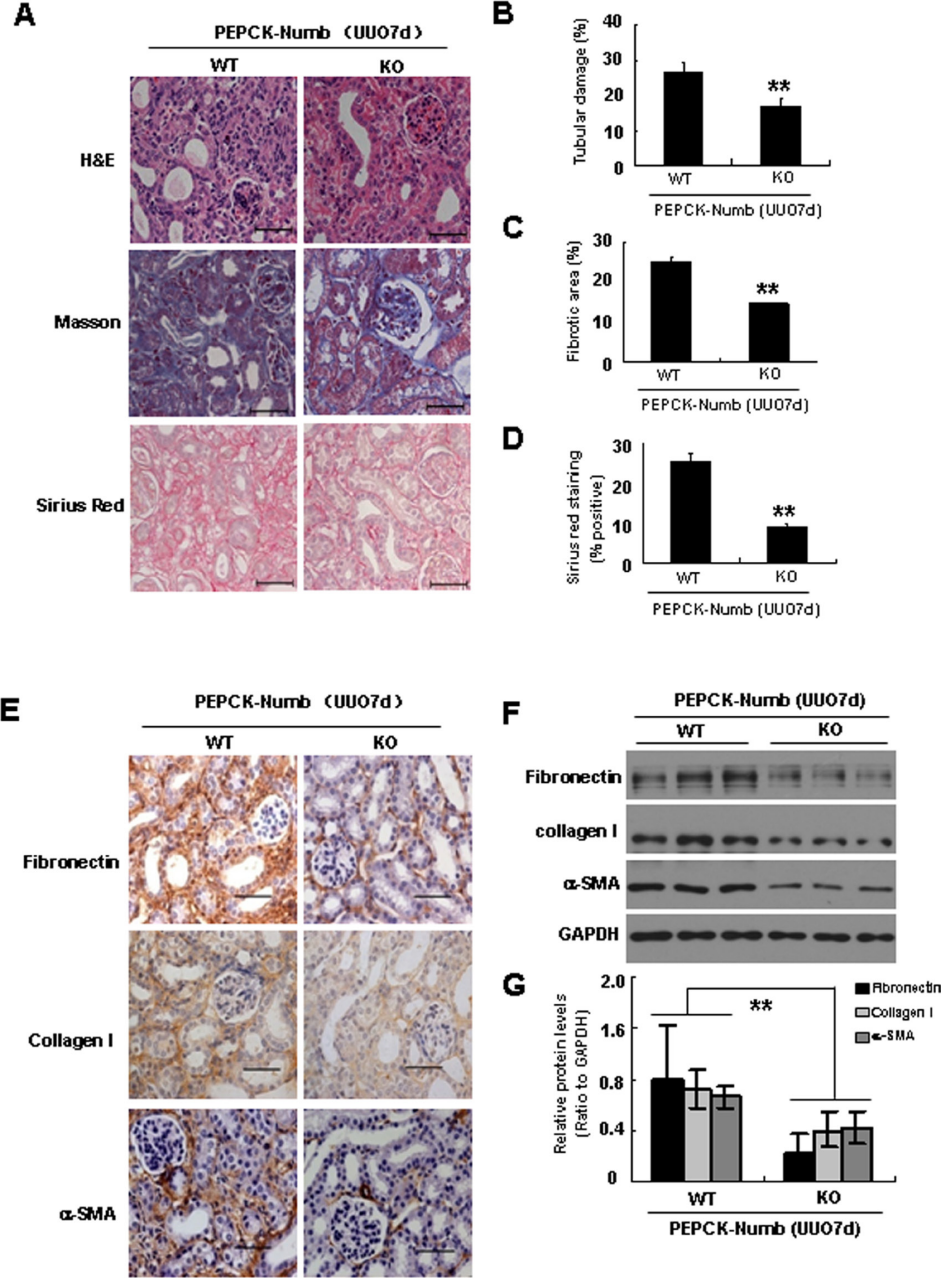
Proximal tubule-specific deletion of Numb ameliorates TIF after UUO **A.** Representative micrographs of H&E, Masson trichrome and picrosirius red staining show morphological changes in the kidneys at day 7 after UUO in PEPCK-Numb-KO and wild type mice. Bar=50μm. **B.** Quantification assessment of tubular dilation and atrophy on the basis of H&E staining. **C.** Quantitative assessment of interstitial fibrosis on the basis of Masson trichrome staining. **D.** Quantitative assessment of collagen accumulation on the basis of picrosirius red staining. Ten fields (×400 magnification) per kidney were used for counting. **E.** Representative images of immunohistochemical staining shows the expressions of fibronectin, collagen I and α-SMA in the obstructed kidneys of PEPCK-Numb-KO and PEPCK-Numb-WT mice at day 7 after UUO. Bar=50μm. **F.** Representative Western blots show the protein level of fibronectin, collagen I and α-SMA in the kidneys of PEPCK-Numb-KO and PEPCK-Numb-WT mice at day 7 after UUO. GAPDH was used to verify equivalent loading. **G.** Graphic representation of relative level of fibronectin, collagen I and α-SMA normalized to GADPH. Data are expressed as mean±SD, n=6. **p<0.01 versus WT UUO.

We next examined the effect of tubule-specific ablation of Numb on TIF in the UUO model. In wild type mice, at day 7 after UUO, kidneys developed severe tubular damage, seen as tubular dilation and atrophy, flattering of tubular cells, increased thickness of the tubular basement membrane, and increase in interstitial space. However, the tubular damage was dramatically attenuated in PEPCK-Numb-KO mice (Figure 3A–3B). In addition, Masson trichrome and picrosirius red staining showed that, compared with wild type mice, interstitial extracellular matrix accumulation was significantly decreased in PEPCK-Numb-KO mice (Figure 3A, 3C-3D). Immunohistochemical staining demonstrated that the abundance of α-SMA, fibronectin, and collagen I protein was significantly lower in the kidneys of PEPCK-Numb-KO mice at day 7 after UUO (Figure 3E). Quantitative determinations by using Western blot analyses of whole kidney lysates produced similar results (Figure 3F-3G).

### Ectopic expression of Numb causes proximal tubular cell arrest at G2/M *in vitro*

**Figure 4:**
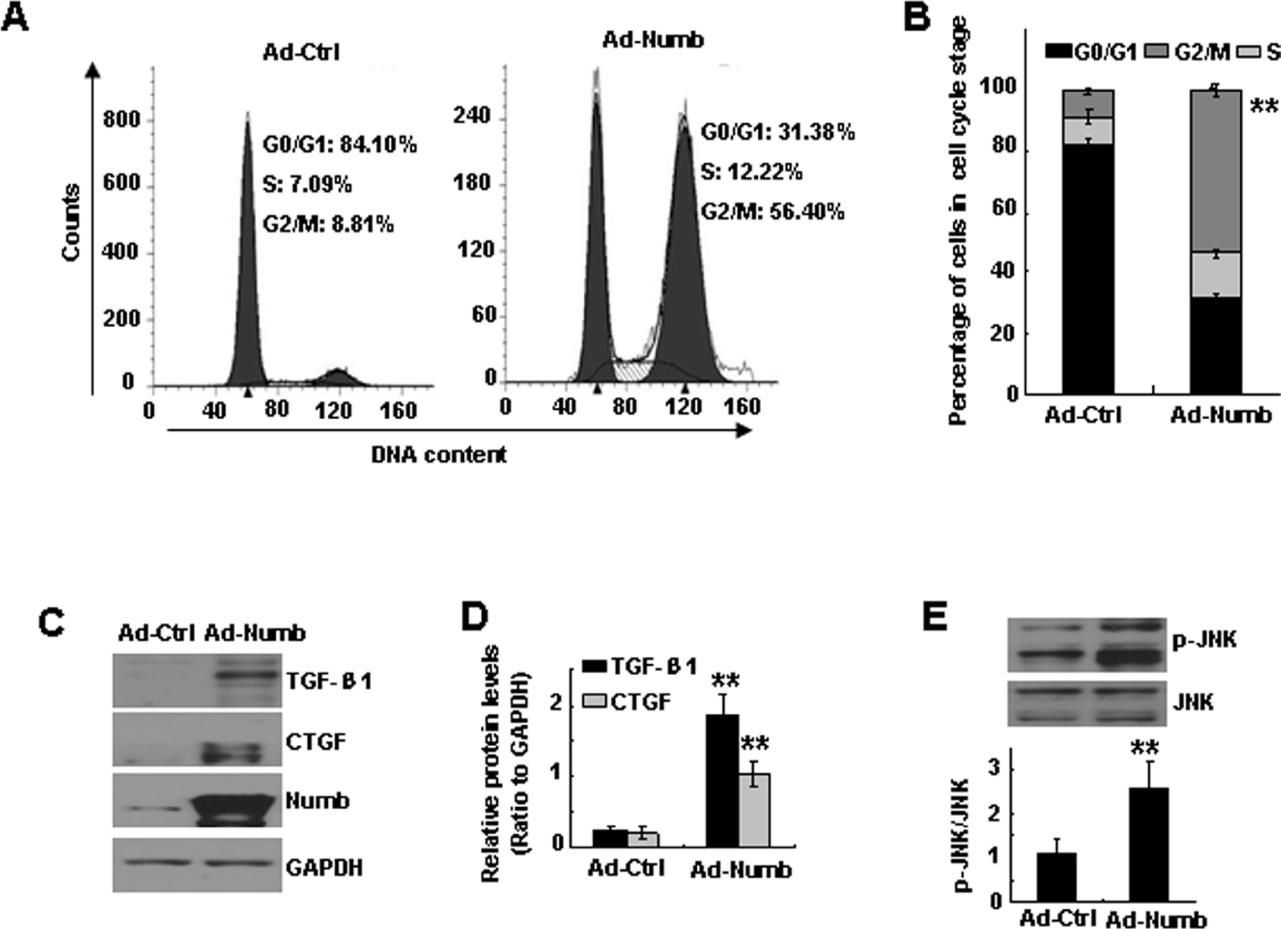
Ectopic expression of Numb causes proximal tubular cell arrest at G2/M *in vitro* HK-2 cells were infected with Ad-Numb or empty vector (Ad-Ctrl). **A.** Cell cycle analysis by propidium iodide staining and flow cytometry in HK-2 cells. **B.** Bar graph depicted the percentage of cells in the different stages of the cell cycle. Symbols in the cell cycle data panels refer to the comparison of G2/M phases. **C.** Western blot analysis of the expression of Numb, TGF-β1, CTGF in Ad-Numb or Ad-Ctrl infected cells. GAPDH was used to verify equivalent loading. **D.** Graphic representation of relative abundance of TGF-β1 and CTGF to GAPDH. **E.** Western blot analysis of p-JNK in Ad-Numb or Ad-Ctrl infected cells (upper panel). Graphic representation of relative abundance of p-JNK to total JNK (lower panel). Data are expressed as mean±SD of three independent experiments. **p < 0.01 versus Ad-ctrl.

Cell cycle arrest of proximal tubular cells at G2/M phase contributes to the pathogenesis of TIF [13], we therefore investigated whether Numb affects cell cycle. To address this issue, Numb was overexpressed in HK-2 cells by infecting with a Numb adenovirus (Ad-Numb). Flow cytometry analysis demonstrated that overexpressing Numb significantly increased the G2/M population of HK-2 cells from 8.81% to 56.40% (Figure 4A-4B), but did not cause obvious apoptosis (Supplementary Figure S4).

It has been reported that profibrotic factors are upregulated in G2/M-arrested tubular cells via activation of JNK signaling [13]. We thus assessed the expression of TGF-β1 and CTGF and the level of phosphorylated JNK (p-JNK). Western blotting showed that the protein level of p-JNK, TGF-β1 and CTGF was significantly increased in Ad-Numb infected cells (Figure 4C-4E). Together, it is concluded that Numb induction causes proximal tubular cells arrest at G2/M phase, and increases the production of profibrotic cytokines.

### Numb causes proximal tubular cell arrest at G2/M through p53 *in vitro*

**Figure 5:**
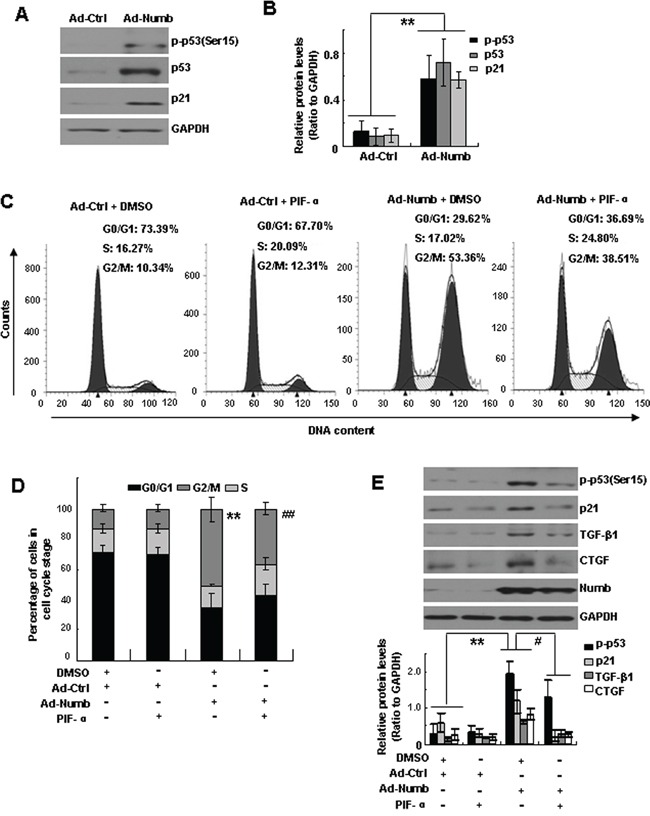
Numb-induced G2/M arrest of proximal tubular cells is mediated by p53 **A.** HK-2 cells were infected with Ad-Numb or Ad-ctrl. Western blotting was performed to analyze the expression of phosphorylated p53 (p-p53), total p53 and p21. GAPDH was used to verify equivalent loading. **B.** Graphic representation of relative abundance of p-p53, p53 and p21 to GAPDH. Data are expressed as mean±SD of three independent experiments. **p < 0.01 versus Ad-ctrl. **C.** HK-2 cells were incubated with Ad-Numb or Ad-ctrl for 24 hours and then treated with PIF-α (20μM) for 48 hours. The same amount of DMSO was used as vehicle control. Cell cycle profiles were determined by flow cytometric analysis. **D.** Bar graph depicted the percentage of cells in the different stages of the cell cycle. Data are expressed as mean±SD of three independent experiments. Symbols in the cell cycle data panels refer to the comparison of G2/M phases. **p<0.01 versus DMSO-treated Ad-ctrl-infected cells. ^##^*p* < 0.01 versus DMSO-treated Ad-Numb-infected cells. **E.** Western blot analysis of the expression of Numb, p-p53, p21, TGF-β1 and CTGF. GAPDH was used to verify equivalent loading. Data are mean±SD of three independent experiments. **p<0.01 versus DMSO-treated Ad-ctrl-infected cells. ^#^*p*<0.05 versus DMSO-treated Ad-Numb-infected cells.

It is well known that p53 is a critical factor regulating cell cycle at the G2 checkpoint in response to stress and DNA damage [24, 25]. Previous studies demonstrated that Numb prevents p53 degradation by disrupting MDM2-p53 complex [19, 26]. We thus hypothesized that Numb promotes cell cycle arrest via stabilizing p53. To address this issue, we first examined the protein level of p53 in Numb overexpressed cells by Western blotting. As expected, the levels of total p53 and phosphorylated p53 (p-p53) were both significantly increased in Ad-Numb infected cells. Moreover, the expression of p21, a transcriptional target of p53, was significantly increased in Ad-Numb infected HK-2 cells (Figure 5A-5B), suggesting the activation of p53-p21 pathway in Numb overexpressed cells.

To examine the role of p53 in Numb-induced G2/M arrest, Ad-Numb infected HK-2 cells were incubated with pifithrin-α (PIF-α), a p53 inhibitor, for 48 hours. Flow cytometry analysis revealed that the fraction of cells in G2/M phase was dramatically reduced by PIF-α treatment (Figure 5C-5D). Correspondingly, Numb-augmented expressions of p-p53, p21, TGF-β1 and CTGF were suppressed by PIF-α treatment (Figure 5E). These data indicated that Numb-induced G2/M arrest of proximal tubular cells is mediated by p53.

### Depletion of Numb attenuates G2/M arrest of proximal tubular cells in obstructive nephropathy

**Figure 6:**
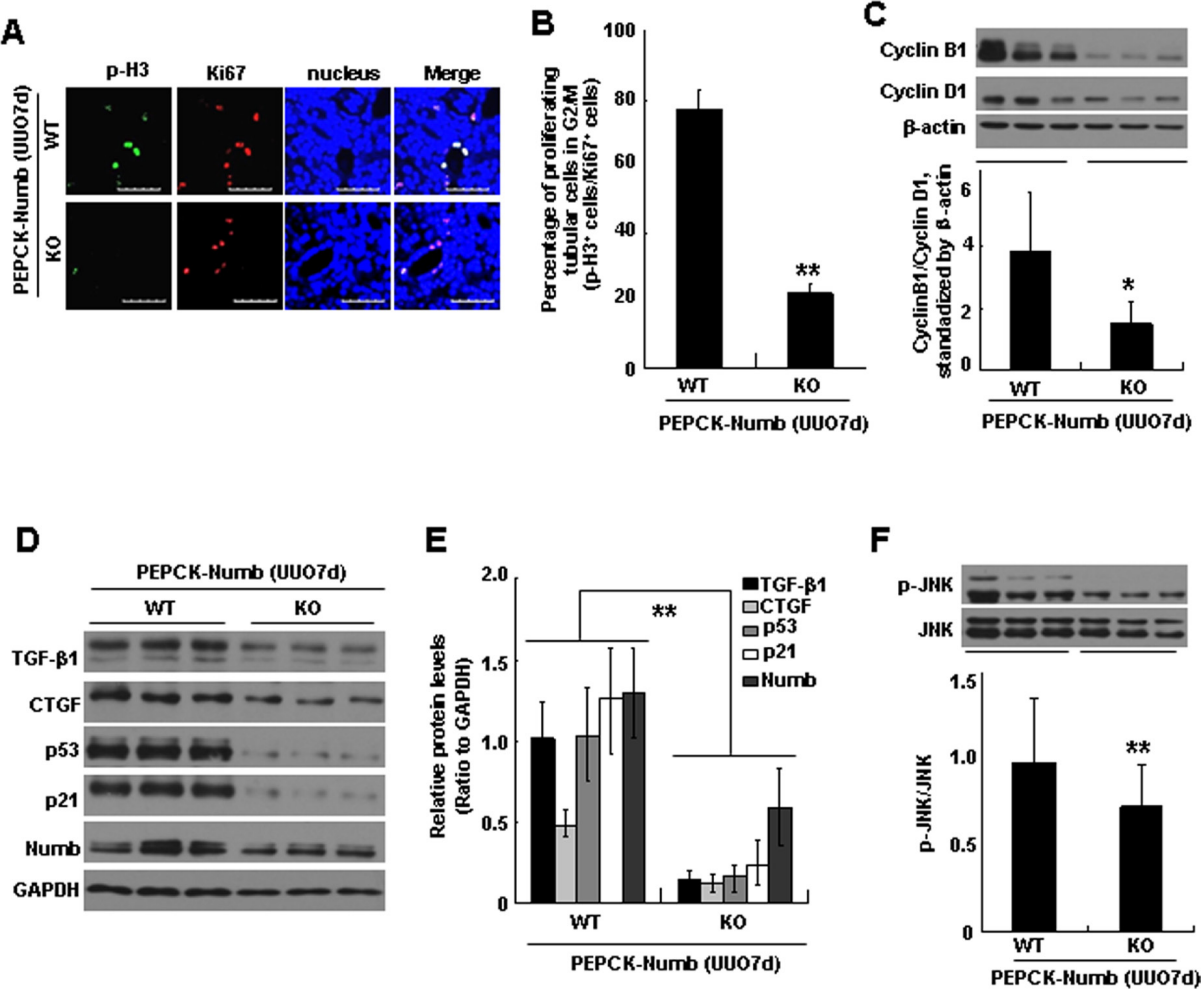
Loss of Numb in proximal tubules reduces G2/M arrest after UUO **A.** Immunofluorescence staining was performed to detect the number of proliferating tubular cells arrested at G2/M phase at day 7 after UUO. Fibrotic kidney sections dissected from PEPCK-Numb-KO and PEPCK-Numb-WT mice were co-stained with p-H3 (green) and Ki67 (red). Nuclei are visualized with DAPI (blue). Bar=50μm. **B.** Bar graph depicted the percentage of proliferating tubular cells (Ki67-positive) in the G2/M phase in the UUO model. **p<0.01 versus WT UUO (n=3). **C.** Western blot analysis of cyclin D1 and cyclin B1 in fibrotic kidney from PEPCK-Numb-KO and PEPCK-Numb-WT mice (top) and ratio of cyclin B1 to cyclin D1 densities standardized to β-actin (bottom). *p<0.05 versus WT UUO (n=6). **D.** Representative Western blots show the protein levels of p53, p21, TGF-β1 and CTGF at day 7 after UUO in PEPCK-Numb-KO and PEPCK-Numb-WT mice. **E.** Graphic representation of relative protein level of p53, p21, TGF-β1 and CTGF normalized to GADPH. **p < 0.01 versus WT UUO (n=6). **F.** Representative Western blot and quantified data for the level of p-JNK in PEPCK-Numb-WT and PEPCK-Numb-KO kidneys at day 7 after UUO. **p < 0.01 versus WT UUO (n=6). Total JNK was used to verify equivalent loading.

We next evaluated the effect of Numb depletion on G2/M arrest of proximal tubular cells in UUO model. The proliferating tubular cells in G2/M phase were detected by co-staining of phosphorylated histone H3 at ser10 (p-H3), a marker for the G2/M phase, with Ki67 (Figure 6A). Compared with wild type mice, the percentage of proliferating tubular cells in G2/M phase was significantly reduced in PEPCK-Numb-KO mice (Figure 6B). Reduction of cells in G2/M phase was further confirmed by the decreased cyclin B1/cyclin D1 ratio (Figure 6C). In addition, Western blotting showed that the expression of p53, p21, TGF-β1, CTGF and the level of p-JNK were dramatically decreased in PEPCK-Numb-KO mice after UUO (Figure 6D-6F).

### Depletion of Numb ameliorates interstitial fibrosis induced by unilateral ischemia reperfusion injury

**Figure 7:**
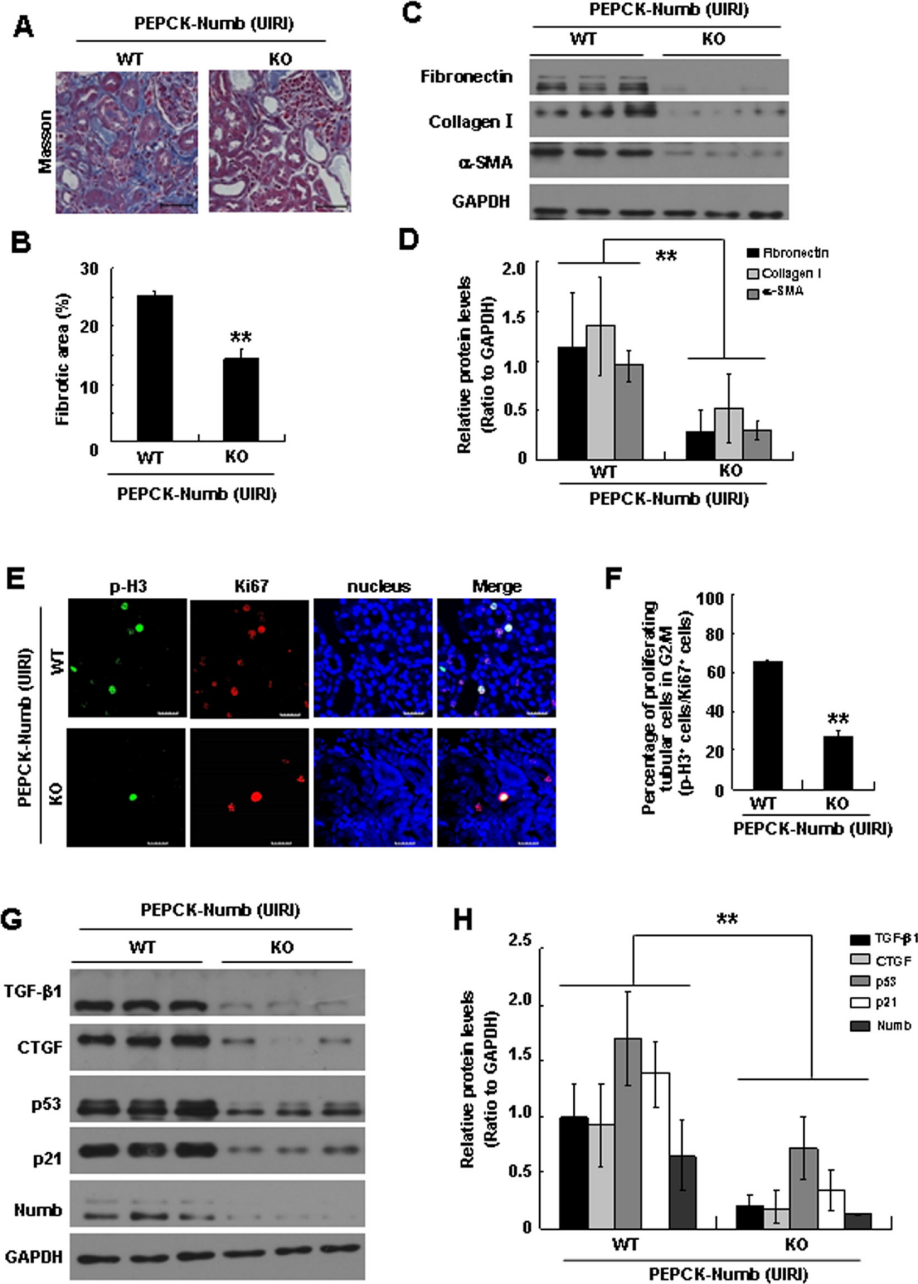
Proximal tubule-specific deletion of Numb ameliorates TIF after UIRI **A.** Renal histologic changes at day 14 after UIRI were assessed by Masson trichrome staining. Bar=50μm. **B.** Histologic damage in Masson-stained UIRI kidney sections was scored by counting the percentage of tubular interstitial fibrotic score. **p<0.01 versus WT UIRI. **C.** Representative western blots show the protein level of fibronectin, collagen I and α-SMA in the kidneys of PEPCK-Numb-KO and PEPCK-Numb-WT mice at day 14 after UIRI. GAPDH was used to verify equivalent loading. **D.** Graphic representation of relative level of fibronectin, collagen I and α-SMA normalized to GADPH. Data are expressed as mean±SD, n=6. **p<0.01 versus WT UIRI. **E.** Co-staining Ki67 with p-H3 on day-14 kidneys after UIRI. Bar=20μm. **F.** Percentage of proliferating cells (Ki67-positive) that are in the G2/M phase of the cell cycle in the UIRI model. **p<0.01 versus WT UIRI (n=3). **G.** Representative Western blots show the protein levels of p53, p21, TGF-β1 and CTGF at day 14 after UIRI. **H.** Graphic representation of relative protein level of p53, p21, TGF-β1 and CTGF in UIRI-induced kidneys normalized to GADPH. **p<0.01 versus WT UIRI (n=6).

To further confirm the role of tubular Numb in TIF, we used another TIF model induced by unilateral ischemia reperfusion injury (UIRI). Masson trichrome staining demonstrated fewer fibrotic lesions in the kidneys of PEPCK-Numb-KO mice (Figure 7A-7B). Western blotting demonstrated that the expression of α-SMA, fibronectin, and collagen I was significantly suppressed in PEPCK-Numb-KO mice (Figure 7C-7D). These data demonstrated that loss of Numb in proximal tubules attenuates TIF in both UUO and UIRI nephropathy models.

We further examined the proportion of proximal tubular cells in the G2/M phase by co-staining p-H3 with Ki67 after UIRI. As shown in Figure 7E-7F, the percentage of proliferating cells in G2/M phase was significantly reduced in PEPCK-Numb-KO mice. Western blotting showed that the expression of TGF-β1, CTGF, p53, and p21 was markedly suppressed in PEPCK-Numb-KO mice (Figure 7G-7H). Collectively, these data indicated that ablation of Numb from proximal tubular cells inhibits G2/M arrest in both UUO and UIRI nephropathy models.

### Numb is upregulated in human fibrotic kidney

**Figure 8:**
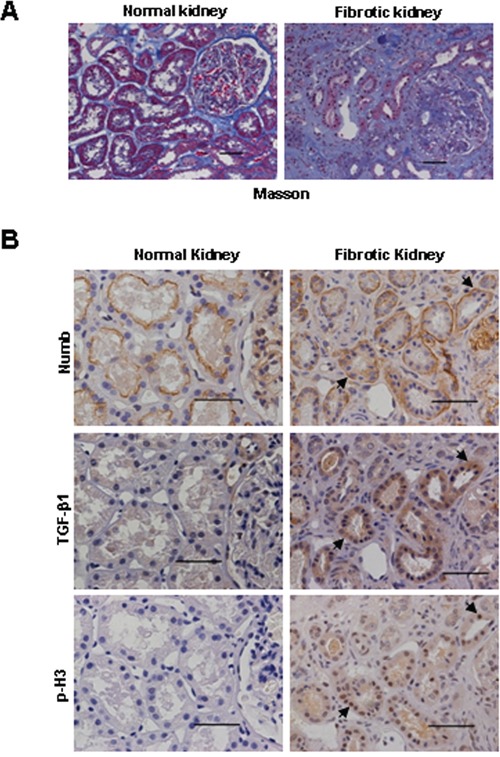
Increased expression of Numb in human fibrotic kidney **A.** Representative micrographs show the collagen deposition detected by Masson trichrome staining in human normal and fibrotic kidneys. Bar=50μm. **B.** Representative images of the immunohistochemical staining of Numb, TGF-β1 and p-H3 in sequential sections of renal biopsies from patients with renal tubulointerstitial fibrosis. Normal renal tissues adjacent to tumor (renal cell carcinoma) were used as normal control. Arrows indicate the co-localization of Numb, TGF-β1 and p-H3 in the injured tubules. Bar=50μm.

To evaluate the clinical relevance of Numb-caused G2/M arrest of tubular cells, we examined the expression of Numb, p-H3 and TGF-β1 in human renal fibrosis biopsies by immunohistochemical staining in sequential sections of renal biopsies from CKD patients. Normal kidney tissues adjacent to the tumor were served as controls. Masson trichrome staining showed a dramatic increase of interstitial collagen deposition in human fibrotic kidney (Figure 8A). As shown in Figure 8B, increased Numb expression was detected in fibrotic renal tissue. Most importantly, Numb was co-localized with p-H3 and TGF-β1 in the injured tubules. These data suggest that dysregulation of Numb is implicated in the pathogenesis of human interstitial fibrosis.

## DISCUSSION

Numb is a highly conserved and ubiquitously expressed adaptor protein. However, the expression and function of Numb in kidney remains largely unknown. Herein, we demonstrate that Numb is expressed in tubules and glomeruli in both mouse and human adult kidney. The expression of Numb was dramatically increased in fibrotic kidney induced by UUO and human fibrotic kidney. Depleting Numb from proximal tubules significantly attenuated TIF induced by UUO and UIRI. These data are, to our best knowledge, the first to characterize a pathogenic role of Numb in the progression of TIF.

Numb is a multifunctional protein involved in many signaling pathways including stabilizing p53 protein [19], antagonizing Notch signaling [27], and mediating transmembrane protein endocytosis and trafficking [28–32]. Our present study demonstrates that Numb provokes G2/M arrest of proximal tubules via stabilizing p53 protein. We and others have reported that Numb inhibits puromycin aminonucleoside- and albumin-induced proximal tubular cells apoptosis [33, 34]. Notably, Numb inhibited puromycin aminonucleoside-induced apoptosis through antagonizing Notch signaling but independent of p53 [33]. To determine the role of Notch signaling in Numb-induced cell cycle arrest, we examined the expression of Notch signaling components and found that the basal protein level of Jagged-1, NICD and Hes-1 protein was pretty low in tubular cells. Overexpression of Numb in proximal tubular cells did not further decrease their expression (Supplementary Figure S3), suggesting that Notch signaling is not involved in Numb-induced G2/M arrest. We speculate that the opposite role of Numb in cell cycle arrest and apoptosis might be due to the distinct signaling pathways involved in these two models.

Many studies have demonstrated a critical role of p53 in various renal injury models [24, 35–37]. Therefore, it is important to understand the mechanism by which the expression and activity of p53 is regulated during the process of renal injury. Posttranslational modifications including phosphorylation and ubiquitination are important for p53 activity. In aristolochic acid nephropathy, aristolochic acid induces dephosphorylation of STAT3 and the subsequent phosphorylation of p53 [37]. The protein stability of p53 is regulated by Murine double minute 2 (Mdm2), an E3 ligase. Mdm2 uses a dual-site mechanism involving both the N terminus and acidic domains to interact with p53, and promote p53 ubiquitination and degradation [38]. Numb is another substrate of Mdm2 and interacts with Mdm2 by the same dual-site mechanism. By binding to both domains on Mdm2, Numb disrupts the Mdm2-p53 complex and Mdm2-catalyzed ubiquitination of p53 [26]. Here, we provided evidence that, in the progression of TIF, the steady state of p53 protein is maintained by induction of Numb expression.

Numb expression was mainly detected in tubules in normal kidney. In fibrotic kidney, Numb expression was also detected in the interstitial cells (Figure 1F). Numb has been shown to be expressed in T cells, fibroblast and macrophage. However, T cell development and T cell activation occur normally in the absence of Numb [39]. In addition, Numb inhibits Sonic Hedgehog/Gli1 signaling activation which is required for fibroblast and macrophage activation [20, 40, 41]. Therefore, we speculate that Numb might not be involved in the activation of T cells, fibroblast or macrophage during the development of TIF.

It has been shown that, Numb and Numbl are expressed together and play redundant roles for the renewal of cardiac progenitor cells [21, 22] and maintaining the polarity of radial glial cells during neocortical neurogenesis [23]. However, in mouse kidney, Numbl, unlike Numb, is predominantly expressed in the glomeruli but not in tubules (Supplementary Figure S1). Moreover, the expression of Numbl did not change dramatically after UUO (Supplementary Figure S2). The different spatial expression patterns of Numb and Numbl suggest that they might play distinct roles in kidney. Further analysis is required to delineate their functions.

**Figure 9:**
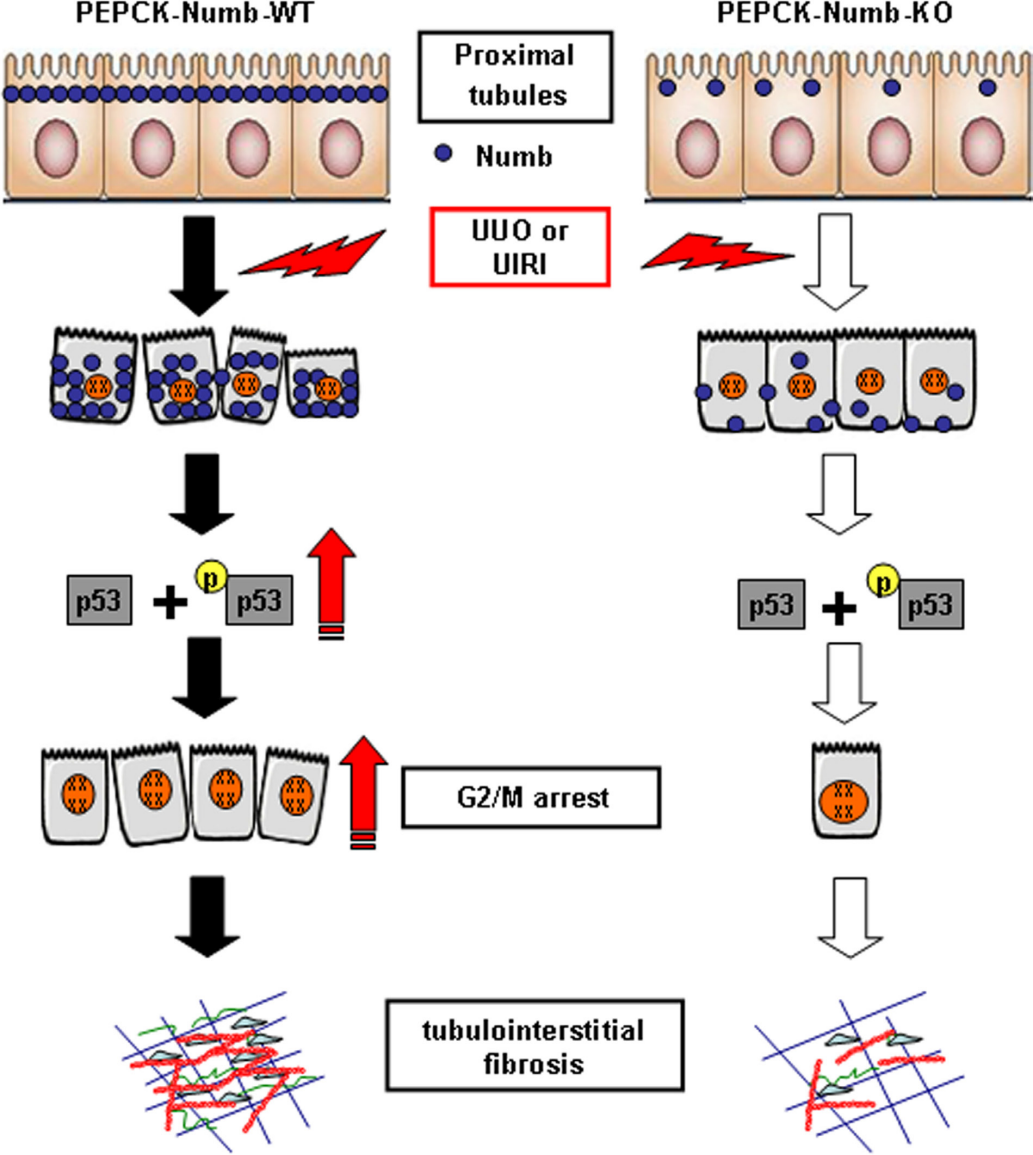
Schematic model depicts the mechanism by which Numb promotes TIF In wild type mice, the expression of Numb in TECs is induced after UUO or UIRI. Increased Numb expression leads to G2/M arrest of proximal tubular cells through stabilizing p53 protein, which in turn induces the expressions of TGF-β1 and CTGF and potentiates renal fibrosis. Tubule-specific knockout Numb reduces the number of proximal tubular cells arrested at G2/M phase (empty arrow), attenuates TIF induced by UUO or UIRI.

In conclusion, our findings, summarized in Figure 9, demonstrate that induction of Numb expression in proximal tubules provokes cell cycle arrest at G2/M phase and thus contributes to TIF. Therefore, targeting Numb might be a novel therapeutic approach for the treatment of fibrotic kidney diseases.

## MATERIALS AND METHODS

### Mouse strains and genotyping

Numb^flox/flox^Numblike^flox/^^+^ mice (C57BL/6J background) were purchased from the Jackson Laboratory (stock number 005384; www.jax.org/index.html). Transgenic mice that expressed Cre recombinase under the control of a modified PEPCK promoter (PEPCK-Cre) was reported elsewhere [42]. Numb^flox/flox^Numblike^+/+^ mice were generated by mating Numb^flox/flox^Numblike^flox/+^ mice. Numb^flox/flox^Numblike^+/+^ mice were intercrossed to PEPCK-Cre mice to produce Numb^flox/flox^Numblike^+/+^ X^CRE^Y (PEPCK-Numb-KO) as depicted in the breeding protocol in Figure 2A. PEPCK-Numb-KO mice were Numb-deleted in proximal tubules. These mice and wild-type littermates (Numb^flox/flox^Numblike^+/+^ XY) were used for experiments. A routine PCR protocol was used for genotyping of tail DNA samples with the following primer pairs: Cre transgene, 5′-GCATAACCAGTGAAACAGCATTGCTG-3′ and 5′-GGACATGTTCAGGGATCGCCAGGCG-3′, which generated a 286bp fragment; Numb genotyping, NumbF (5′-GAAGGAGCCTTCCAAAATCGTATTC-3′) and NumbR (5′-AGGCTTCTGGGAAACCTCACTTACTC-3′), which yielded a 400bp band for the floxed alleles. Numb deletion in renal cortical tissues was confirmed by using primers NumbDel (5′-AGCTTACAGGGTGCCCCAGTTACTA-3′) and NumbR for a 700-bp deletion allele product. All animals were housed in the Nanfang Hospital Animal Center and were born normally at the expected Mendelian frequency. The mice were normal in size and did not display any gross physical or behavioral abnormalities. All animal experiments were performed according to a protocol approved by Ethics Committee for Animal Experiments of the Southern Medical University.

### Mouse models of renal fibrosis

UUO was performed using an established protocol as described previously [43]. Briefly, the left ureter was ligated twice using 4-0 nylon surgical sutures at the level of the lower pole of kidney. Mice were killed at day 3, 7 or 14 after surgery. Serum and kidney samples were collected for various analyses.

UIRI was induced by the retroperitoneal approach on the left kidney for 30 min at 37°C. One milliliter of warm saline (37°C) was injected intraperitoneally after surgery for volume supplement. Sham operations were performed with exposure of left kidney but without induction of ischemia. Mice were killed at day 14 after surgery. Serum and left kidney samples were collected for various analyses.

### Virus construct and infection

E1 and E3 regions deficient serotype 5 recombinant adenovirus vector that tagged with hemagglutinin sequence and contained a cytomegalovirus (CMV) promoter driving mouse cDNA fragment encoding Numb (Ad-Numb) was constructed and generated by SinoGenoMax Co. Ltd (www.sinogenomax.com, Beijing, China). The Ad-CMV-HA (Ad-ctrl) was used as control.

When reached 60% confluences, HK-2 cells were incubated with Ad-Numb or Ad-ctrl for 24 hours and then adenovirus was washed out. Cells were further grown in DMEM/F12 medium supplemented with 2% FBS for 48 hours before proceeding in the data analysis. To observe the role of p53 in Numb-induced cell cycle arrest, pifithrin-α (20μM, Sigma-Aldrich) was added into the medium after infection.

### Flow cytometry analysis

HK-2 cells were washed with cold PBS and then stained with the Cycletest™ Plus DNA Reagent Kit (Becton Dickinson, CA, USA) according to the manufacturer's instructions. Cell cycle distribution was evaluated using a FACSCanto II Flow cytometry (Becton Dickinson, CA, USA). The results were analyzed using the ModFit LT3.3 software. For each experiment, 10000 events per sample were recorded.

Apoptosis was detected by using PE Annexin V apoptosis detection kit (Becton Dickinson, USA). HK-2 cells were incubated with a mixture of PE Annexin V and 7-Amino-Actinomycin (7-AAD), and apoptosis was measured by flow cytometry.

### Real-time PCR

Total RNA was isolated from kidney tissues using TRIzol reagent, according to the manufacturer's instructions (Invitrogen, Carlsbad, CA). Real-time RT-PCR was performed on an ABI PRISM 7500 Fast sequence detection system (Applied Biosystems, Foster City, CA) as described previously [44]. The primer pair for Numb was 5′- CAACACTGCTCCATCCCCAT -3′ and 5′- AATCCCCGGAAAGAGCCTTG -3′. The mRNA levels of various genes were calculated after normalizing with GAPDH by the comparative CT method (2^−DDCT^).

### Western blot analysis

Cells and kidney tissues were lysed with RIPA buffer (1% sodium deoxycholate, 0.1% SDS, 1% Triton X-100, 10mM Tris pH 8.0 and 140mM NaCl, 1% NP-40, 0.1% SDS, 100 mg/ml PMSF, 1% protease inhibitor cocktail, and 1% phosphatase I and II inhibitor cocktail), and lysates were subjected to Western blot analysis using method described previously [44]. The following primary antibodies were used in this study: anti-α-SMA and anti-fibronectin (Sigma-Aldrich, St. Louis, MO, USA); anti-collagen I (Calbiochem, EMD Biosciences, Darmstadt, Germany); anti-p53, anti-p21 and anti-cyclinB1 (Santa Cruz Biotechnology, CA, USA); anti-JNK, anti-p-JNK, anti-p-p53, anti-TGF-β1, anti-Numb and anti-cyclinD1 (Cell Signaling Technology, Beverly, MA, USA); anti-CTGF (Abcam, Cambridge, UK).

### Immunofluorescence and immunohistochemical staining

Immunofluorescence staining was performed using an established procedure [13]. Briefly, paraffin fixed kidney sections were rehydrated and incubated with primary antibody overnight at 4°C, followed by incubation with secondary antibody conjugated with Alexa Fluor 488 or 588 (Molecular Probes, Inc., Eugene, OR, USA). The slides were then counterstained with 49, 6-diamidino-2-phenylindole (DAPI) to visualize the nuclei. Images were taken by confocal microscopy (Olympus, Tokyo, Japan). Antibodies used in this study include: anti-p-H3 (Ser10) (Abcam, Cambridge, UK), anti-Numb (Cell Signaling Technology, Beverly, MA, USA), anti-megalin (Abcam, Cambridge, UK) and anti-Ki67 (Abcam, Cambridge, UK).

Immunohistochemical staining was performed on 4μm kidney sections as previously described [43, 44]. Briefly, sections were deparaffinized and rehydrated in ethanol. After antigen repairing, sections were incubated with the primary antibody against Numb (Cell Signaling Technology, Beverly, MA, USA), p-H3(Ser10) and TGF-β1 (Abcam, Cambridge, UK), α-SMA and fibronectin (Sigma-Aldrich Sigma-Aldrich, St. Louis, MO, USA), Collagen I (Calbiochem, EMD Biosciences, Darmstadt, Germany) for 14 h at 4°C, followed by incubating with secondary antibodies (Dako, Carpinteria, CA) for 30 min. Images were taken by an Olympus BX51 microscope (Olympus, Tokyo, Japan). The average integrated optical density (IOD) and the positive area of Numb staining were determined using image analysis software (Image-Pro Plus 6.0, Media Cybernetics, Silver Spring, MD, USA) by scanning 10 non-overlapping fields at ×400 magnification for each kidney section. Numb-positive area was presented as a percentage of total area.

### Histology

2μm paraffin-embedded kidney sections were subjected to Masson trichrome or H&E staining using commercial kits (Sigma-Aldrich, St. Louis, MO, USA) according to the manufacturer's protocol. 4μm sections were cut for picrosirius red staining. Picrosirius red staining was performed using 0.1% picrosirius red (Direct Red 80, Sigma) and counterstained with Weigert's hematoxylin.

A standard point counting method was used to determine the score of tubular dilation and atrophy [45]. Briefly, under high magnification (×400), 10 non-overlapping fields from each section of the renal cortex were photographed. A grid containing 100 (10×10) sampling points was superimposed on each photograph. Points falling on glomerular structures or on large vessels were excluded from the total count. The tubular dilation and atrophy score was determined by the number of points overlying dilated or atrophied tubular spaces and then converted to a percentage.

For the analysis of the interstitial fibrosis in mice, ten 400× visual fields were randomly selected for each Masson trichrome and picrosirius red stained kidney section and interstitial fibrosis was manually evaluated by a background subtraction method using Image-Pro Plus 6.0 (Media Cybernetics, Silver Spring, MD, USA). Briefly, ten non-overlapping bright field images of renal cortex were captured and the integrated optical density of the positive staining was determined. Quantification (n=6 per group) is presented as the percentage of the ratio of optical density of positive staining to the entire spectrum of a given image.

### Determination of serum creatinine, urinary albumin and urinary creatinine

Urine albumin was measured by using a mouse Albumin ELISA Quantitation kit, according to the manufacturer's protocol (Bethyl Laboratories, Inc., Montgomery, TX, USA). Urine creatinine was determined by a routine procedure as described previously [46]. Urinary albumin was standardized to urine creatinine and expressed as μg/mg urinary creatinine. Serum creatinine level was determined by use of the QuantiChrom creatinine assay kit (BioAssay Systems, Hayward, CA, USA) according to the manufacturer's protocol. The level of serum creatinine was expressed as microgram per deciliter.

### Human kidney specimens

Kidney biopsies samples were obtained from patients with chronic renal tubulointerstitial fibrosis. Normal kidney tissues adjacent to renal carcinoma obtained from nephrectomies were used as controls. 2.0μm paraffin-embedded sections were prepared using a routine procedure. The study was approved by Medical Ethics committee of Southern Medical University. Written informed consent was obtained from all patients.

### Statistical analyses

Data were expressed as means ± SD. Comparisons between two groups were conducted using the two-tailed *t* test. Differences among more than two groups were compared using one-way ANOVA. *P*<0.05 was considered statistically significant.

## SUPPLEMENTARY FIGURES


