# Supplementary material for: Numb contributes to renal fibrosis by promoting tubular epithelial cell cycle arrest at G2/M
Source: Oncotarget. 2016 Mar 21;7(18):25604–19. doi: 10.18632/oncotarget.8238 (PMC5041930; doi:10.18632/oncotarget.8238)
Supplement: Supplementary file 1 [file oncotarget-07-25604-s001.pdf]

SUPPLEMENTARY FIGURES

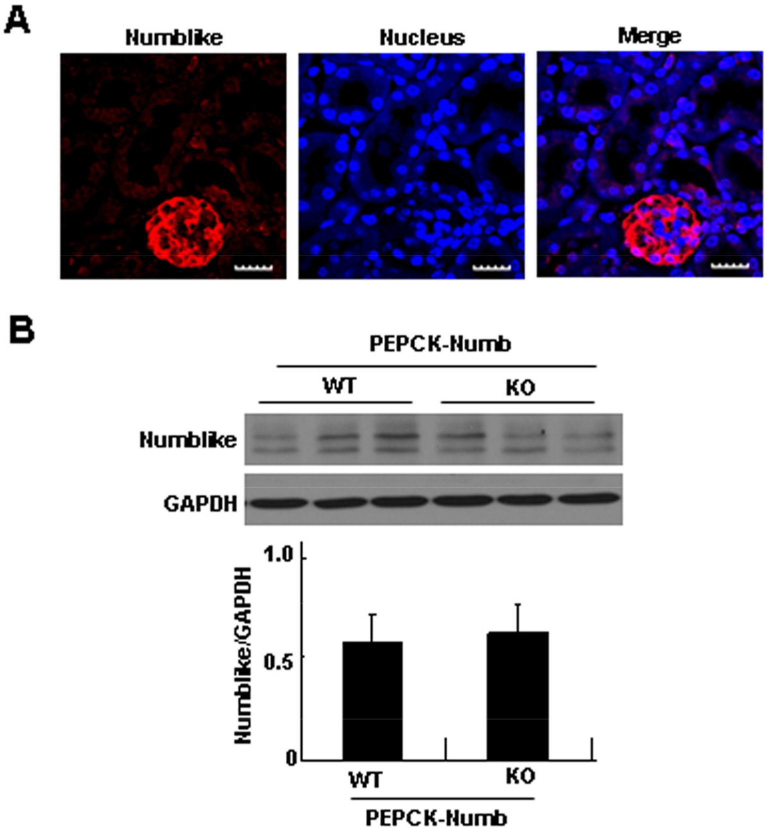

Supplementary Figure S1: Expression and distribution of Numblike in kidney. A. Immunofluorescence staining by using anti-Numblike antibody shows that Numblike protein was mainly detected in the glomeruli in normal adult kidney of C57BL/6J mice at the age of 8-12 weeks. Bar=20µm. **B.** Representative western blots show the protein level of Numblike in PEPCK-Numb-KO and wild type mice.

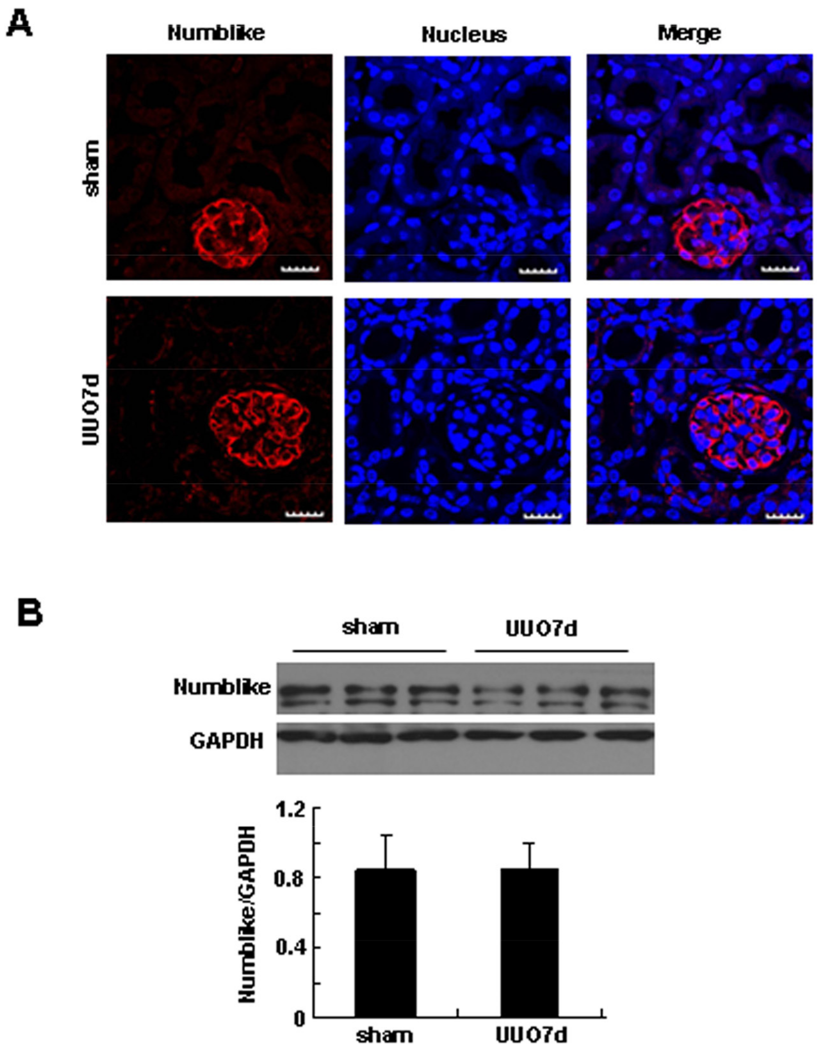

Supplementary Figure S2: Expression and distribution of Numblike in kidney. A. Immunofluorescence staining by using anti-Numblike antibody shows that the expression of Numblike did not change dramatically after UUO. Bar=20μm. **B.** Representative western blots show the protein levels of Numblike in fibrotic kidney at day 7 after UUO.

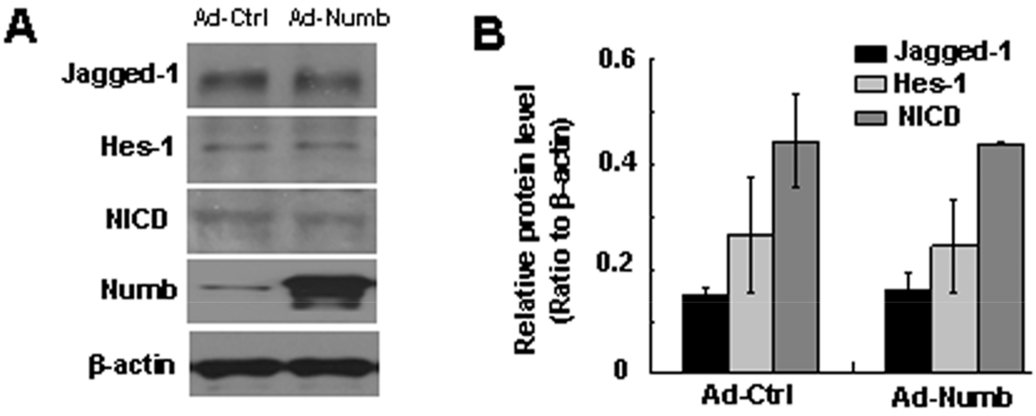

Supplementary Figure S3: Overexpressing Numb did not change the expressions of Notch signaling components. **A.** Representative western blots show that overexpressing Numb did not further decrease the expressions of Jagged-1, NICD and Hes-1. **B.** Graphic representation of relative protein levels of Jagged-1, NICD and Hes-1to GAPDH. Data are expressed as mean±SD of three independent experiments.

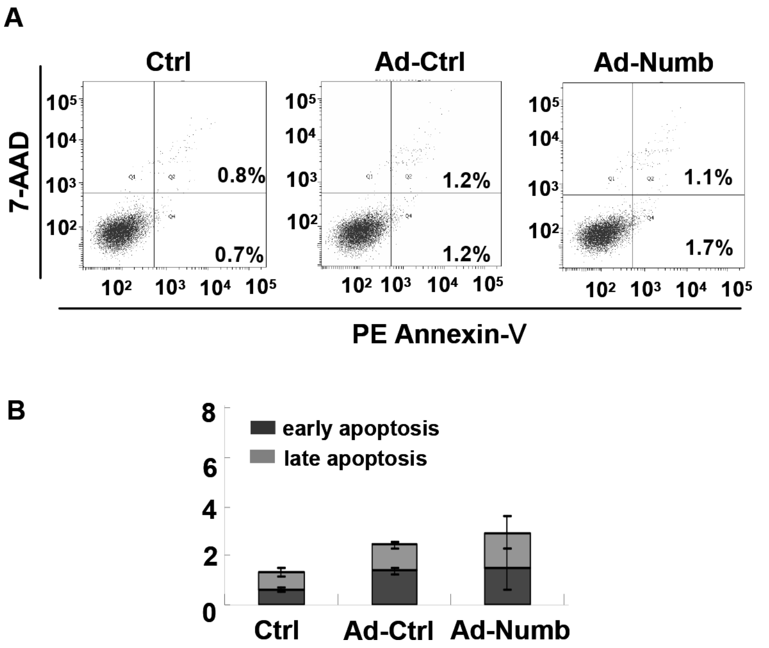

Supplementary Figure S4: Overexpressing Numb did not cause obvious apoptosis in HK2 cells. HK-2 cells were infected with Ad-Numb or Ad-Ctrl. Cell apoptosis was determined by flow cytometric analysis **A.** Quantitative analysis of cell apoptosis by flow cytometry **B.**
